# Complete genome sequence of *Escherichia coli* EPI300

**DOI:** 10.1128/mra.01124-25

**Published:** 2025-12-29

**Authors:** Tony X. Liu, Sean Formby, Beth Davenport, Resmi Capron, Andras Szeitz, Steven J. Hallam

**Affiliations:** 1 Graduate Program in Bioinformatics, University of British Columbia 8166, Vancouver, British Columbia, Canada; 2 Department of Microbiology & Immunology, University of British Columbia 8166, Vancouver, British Columbia, Canada; 3 Genome Science and Technology Program, University of British Columbia 8166, Vancouver, British Columbia, Canada; 4 ECOSCOPE Training Program, University of British Columbia 8166, Vancouver, British Columbia, Canada; 5 Bradshaw Research Institute for Minerals and Mining (BRIMM), University of British Columbia 8166, Vancouver, British Columbia, Canada; 6 Life Sciences Institute, University of British Columbia 8166, Vancouver, British Columbia, Canada; Wellesley College, Wellesley, Massachusetts, USA

**Keywords:** genome, heterologous gene expression, functional screening, single molecule sequencing, model system, plasmid copy control

## Abstract

Here, we report on the complete genome sequence of *Escherichia coli* EPI300. This strain harbors an inducible plasmid copy number control system supporting DNA library production and functional screening applications. PacBio long-read sequencing and assembly resolved a closed circular reference genome useful for mapping and editing the EPI300 genetic operating system.

## ANNOUNCEMENT

*Escherichia coli* EPI300 encodes a genetic operating system developed with plasmid copy control supporting DNA library production and functional screening applications (1–4). The strain was created by a chromosomal insertion into the base strain DH10B (5), also known as EC100, and included a dihydrofolate reductase (*dfrA*) conferring trimethoprim resistance linked to a two-gene cassette sourced from *E. coli* JW366 involved in plasmid copy control (6). This cassette consists of a mutant version of the gene encoding trans-acting replication initiation protein (*trfA203*) under control of an arabinose inducible P_BAD_ promoter accompanied by a gene encoding arabinose regulatory protein (*araC*). The mutant TrfA203 binds to iterons but is incapable of forming higher order structures associated with origin coupling or “handcuffing,” a form of replication control (7, 8). When EPI300 is transformed with a plasmid containing the *oriV* origin of replication recognized by TrfA203, the plasmid copy number can be increased from single copy to 10–100 copies per cell upon L-arabinose addition (6).

A glycerol stock of EPI300 was inoculated in Luria broth and grown for 16 h at 37°C. Approximately 10^9^ cells based on OD_600nm_ were harvested, and genomic DNA was extracted using the NEB Monarch High Molecular Weight DNA Extraction Kit (NEB, T3060S), yielding a final concentration of 5.2 ng/µL. The average DNA fragment length was >60 kb, as measured with a 4150 TapeStation System (Agilent, G2992AA). DNA was sheared by pipette-tip cycling on a Microlab Nimbus (Hamilton), then SMRTbell libraries were prepared using PacBio SMRTbell Prep Kit 3.0 and size-selected on a BluePippin (3–50 kb). Sequencing on the PacBio platform using the Revio Polymerase Kit (HiFi chemistry) generated 265,290 raw reads with a maximum length, N50, and total reads of 37,897 bp, 13,540 bp, and ~3.4 × 10^9^ bp respectively. Filtlong (9) (v0.2.0) was used to obtain the best reads adding up to 5 × 10^8^ bp (~100× coverage) to improve assembly contiguity. *De novo* assembly of Filtlong’s output using Hifiasm-meta (10) (v0.19.8-r603) produced one 4,691,561 bp circular contig. Read and assembly statistics were calculated by seqkit (11) stat (v2.3.0), while the assembly quality was assessed by CheckM (12) (v1.2.2). The NCBI Prokaryotic Genome Annotation Pipeline (13) (PGAP, v6.10) was used to perform automated gene feature prediction and annotation (Table 1). Default parameters were used, unless otherwise noted.

**TABLE 1:** Genome features of *Escherichia coli* EPI300

NCBI accession number	Genome size (bp)	Contigs	GC (%)	Genes	Protein CDS	CRISPR arrays	Pseudogenes	rRNA operons	tRNA operons	Completeness/contamination (%)
CP189566	4,121,253	1	50.78	4,597	4,260	2	215	7	86	98.86/1.82

**Fig 1:**
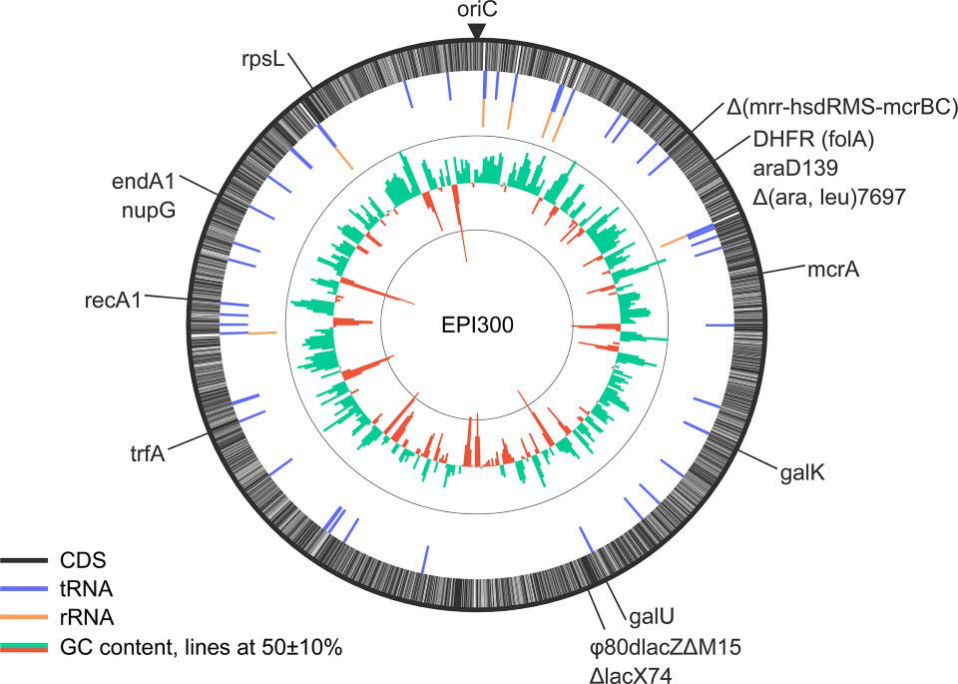
Genome summary of EPI300 based on genotype description.

The EPI300 genotype is reported as *F^-^ mcrA Δ(mrr-hsdRMS-mcrBC) φ80dlacZΔM15 ΔlacX74 recA1 endA1 araD139 Δ(ara, leu)7697 galU galK λ^-^ rpsL nupG trfA dhfr* (14) and confirmed by comparing against the wild-type reference K12 MG6155 (NCBI: U00096.3; Fig. 1). The *trfA* gene-cassette insertion was localized to codon position 121 of a predicted O-antigen modification gene, *yfdH* (locus ACP6EX_16650). Arabinose operon genes *araAB* were not located, while *araD* and both high-affinity *araFGH* and low-affinity *araE* transport systems were identified. Pangenome analysis with PPanGGOLiN (15) (v2.2.4) using a panel of reference strains indicated that EPI300 was most similar to DH10B (NCBI: NC_010473.1), differing by only 14 genes. For the remainder, EPI300 differed by 72, 72, 135, and 1,216 genes when compared to DH5-α (NCBI: NZ_CP076470.1), TOP10 (NCBI: NZ_CP080620.1), K12 MG1655 (NCBI: U00096.3), and O157 (NCBI: BA000007.3), respectively.

## Data Availability

The complete genome of *E. coli* EPI300 is publicly available under NCBI accession no. CP189566 within BioProject PRJNA1255200 and BioSample SAMN48137190. Raw reads can be found in NCBI’s Sequence Read Archive (accession no. SRR35110061).
